# Phospholipid-Coated Mesoporous Silica Nanoparticles Acting as Lubricating Drug Nanocarriers

**DOI:** 10.3390/polym10050513

**Published:** 2018-05-09

**Authors:** Tao Sun, Yulong Sun, Hongyu Zhang

**Affiliations:** State Key Laboratory of Tribology, Department of Mechanical Engineering, Tsinghua University, Beijing 100084, China; sunt26@yeah.net (T.S.); yulong.sun@hotmail.com (Y.S.)

**Keywords:** osteoarthritis, mesoporous silica nanoparticles, phospholipids, hydration lubrication, drug delivery

## Abstract

Osteoarthritis (OA) is a severe disease caused by wear and inflammation of joints. In this study, phospholipid-coated mesoporous silica nanoparticles (MSNs@lip) were prepared in order to treat OA at an early stage. The phospholipid layer has excellent lubrication capability in aqueous media due to the hydration lubrication mechanism, while mesoporous silica nanoparticles (MSNs) act as effective drug nanocarriers. The MSNs@lip were characterized by scanning electron microscope, transmission electron microscope, Fourier transform infrared spectrum, X-ray photoelectron spectrum, thermogravimetric analysis and dynamic light scattering techniques to confirm that the phospholipid layer was coated onto the surface of MSNs successfully. A series of tribological tests were performed under different experimental conditions, and the results showed that MSNs@lip with multi-layers of phospholipids greatly reduced the friction coefficient in comparison with MSNs. Additionally, MSNs@lip demonstrated sustained drug release behavior and were biocompatible based on CCK-8 assay using MC3T3-E1 cells. The MSNs@lip developed in the present study, acting as effective lubricating drug nanocarriers, may represent a promising strategy to treat early stage OA by lubrication enhancement and drug delivery therapy.

## 1. Introduction

Osteoarthritis (OA) is a common chronic joint disease suffered by millions of patients, which is featured by wear and degeneration of articular cartilage [1]. The prevalence rate of OA is relatively high, and it is reported that more than 50% of people aged over 60 could be affected by OA [2]. Basically, the clinical treatment for end stage OA is artificial joint replacement [3,4], but treating OA at an early stage through a non-surgical technique is considered to be more preferable for patients. In terms of treating early stage OA, selected drugs are employed in order to reduce inflammation, such as bisphosphonates [5] and receptor antagonists [6]. However, it can be anticipated that generally it is difficult for the drugs to reach joints partly due to the insufficiency of blood vessels in the articular cartilage, which results in a considerably low absorption rate [7]. Consequently, the use of drug nanocarriers, which can directly be injected into joint capsules for local drug delivery, is considered to be a valuable attempt to address this problem [8]. Mesoporous silica nanoparticles (MSNs) are reported as efficient drug nanocarriers, which can achieve targeted and controlled drug delivery due to their highly ordered mesoporous structure, large surface area and possibility for further modification [9,10,11,12,13]. Nevertheless, the injection of MSNs into joint capsules may also introduce abrasive nanoparticles and even further destroy joint lubrication, which can greatly accelerate the development of OA [14]. Therefore, MSNs should be modified with effective lubricating materials to achieve better lubrication.

Phospholipids are amphiphiles featured by hydrophilic phosphatidylcholine (PC) head groups and hydrophobic hydrocarbon backbones, and are easily self-assembled to form various structures, such as bilayers and vesicles (liposomes). In previous studies, liposomes, which have been detected to be present on the outer surface of articular cartilage [15,16], are proved to enhance lubrication as an efficient boundary lubricant [17,18,19]. The low friction property of liposomes in an aqueous environment is attributed to the hydration lubrication mechanism of the highly hydrated PC head groups exposed at the outer surface [20]. Recently, phospholipid-coated MSNs (MSNs@lip) have received rapidly increasing attention as drug nanocarriers for controlled drug delivery, which benefit from the “protocell” structure mimicking the cell membrane’s property of the impermeability of hydrophilic molecules [21,22]. It is anticipated that the phospholipid layer of MSNs@lip may play a similar role to liposomes upon applied load, acting as an effective boundary lubricant, but this hypothesis has not been reported in previous investigations. Accordingly, in the present study, phospholipid-coated MSNs were synthesized, and the lubrication property, drug loading and release behavior and in vitro cytotoxicity of the nanoparticles were examined by a series of tribological tests, dialysis tube diffusion and CCK-8 assay, respectively, which aimed to verify whether the phospholipid-coated MSNs could be used to treat early stage OA.

## 2. Materials and Methods

### 2.1. Materials

Cetyltrimethyl ammonium bromide (CTAB, 98%), distearoyl phosphatidylcholine (DSPC, 98%) and cholesterol (99%) were purchased from J&K Scientific Co., Beijing, China. NaOH (99%), tetraethyl orthosilicate (TEOS, 98%), chloroform (99%), hydrochloric acid (HCl, 37%) and methanol (99%) were purchased from Beijing Chemical Reagent Co., Beijing, China. Rhodamine B (RhB, 96%) was purchased from Solarbio Co., Beijing, China.

### 2.2. Synthesis of DSPC Liposomes

DSPC (7.9 mg) and cholesterol (1.97 mg) were dissolved in a methanol-chloroform (85:15, *v*/*v*, 2 mL) mixture. The solution was evaporated in a flask using a rotary evaporator at 60 °C, and the phospholipid film remained at the bottom of the flask. Afterwards, the flask was ultrasonically shaken in 2 mL of deionized water and heated at 60 °C for 1 h to generate DSPC liposomes.

### 2.3. Synthesis of MSNs

MSNs were prepared with reference to the procedures of our previously published paper [23]. Briefly, 0.5 g of CTAB and 1.75 mL of NaOH solution (2 M) were added into aqueous solution (240 mL), and the solution was stirred at 80 °C. Subsequently, 5 mL of TEOS was added, and the solution was stirred at 80 °C for 6 h. The resulting product was filtered and washed with sufficient deionized water and methanol. The CTAB template was extracted by stirring in 50 mL of methanol and 5 mL of HCl at 60 °C for 24 h. Finally, the resulting product was centrifuged, washed with methanol and dried under vacuum overnight.

### 2.4. Synthesis of Phospholipid-Coated MSNs

Phospholipid-coated MSNs with a single layer (MSNs@lip-1) were synthesized based on a previous publication [24]. Briefly, the as-prepared MSNs (20 mg) were added into the DSPC liposomes solution (2.5 mg/mL, 10 mL), and then the mixture was placed at 60 °C for 1 h. The resulting product was centrifuged (8000 r/min, 3 min) and dried under vacuum overnight. In order to prepare phospholipid-coated MSNs with multi-layers, 5 mg of MSNs@lip-1 was added uniformly into a flask with phospholipid film. Subsequently, the flask was ultrasonically shaken in 2 mL of deionized water and heated at 60 °C for 1 h. Finally, the resulting product was centrifuged (8000 r/min, 3 min) and dried under vacuum overnight as mentioned above (MSNs@lip-2).

### 2.5. Characterization Analysis

MSNs, MSNs@lip-1, and MSNs@lip-2 were characterized by the following analyses. Scanning electron microscopy (SEM, Quanta 200, FEI, Eindhoven, The Netherlands) and transmission electron microscopy (TEM, JEM-2100F, FEI, JEOL, Tokyo, Japan) were used to examine the morphology of the nanoparticles. Fourier transform infrared spectrum (FTIR) was recorded using a Nexus 670 Spectrometer (Thermo-Nicolet, Madison, WI, USA) with a wavelength of 400 cm^−1^–4000 cm^−1^. An X-ray photoelectron spectrum (XPS) was obtained using an Escalab 250 Xi spectrometer (Thermo Scientific, Waltham, MA, USA), and the binding energy was calibrated against the O1s peak at 523 eV. Thermogravimetric analysis (TGA) was conducted on a Q5000 instrument (TA Instruments, New Castle, DE, USA) at a heating rate of 10 °C/min from 25 °C to 800 °C. Hydrodynamic diameter and Zeta potential were measured by a dynamic light scattering technique (DLS) using a Zetasizer Nano ZS size analyzer (Malvern Instruments, Malvern, UK) equipped with a 633 nm He-Ne laser.

### 2.6. Lubrication Property

The lubrication property of phospholipid-coated MSNs was evaluated through a series of tribological tests. The tribological tests were conducted using a UMT-3 universal material tester (Centre for Tribology Inc., Campbell, CA, USA) operated in a reciprocating mode (oscillation amplitude: 4 mm). Different experimental conditions were attempted as follows: sliding frequency (1 Hz, 5 Hz and 10 Hz); normal load (1 N, 2 N and 4 N); MSNs@lip concentration (2, 5 and 10 mg/mL), test duration (30 min). A polished Ti6Al4V disk was used as lower specimen, which slid against a polyethylene sphere pin (diameter: 8 mm), as the upper specimen. The normal load is equivalent to an apparent maximum contact pressure of 26 MPa (1 N), 32 MPa (2 N), and 41 MPa (4 N), which was calculated based on Hertz theory for ball-on-flat configuration as shown in the following equation, where P is the apparent maximum contact pressure, *F* is the normal load, *R* is the radius of the pin (4 mm), *E*_1_ and *µ*_1_ are the elastic modulus and Poisson’s ratio of Ti6Al4V (110 GPa and 0.3), and *E*_2_ and *µ*_2_ are the elastic modulus and Poisson’s ratio of polyethylene, respectively (1 GPa and 0.4) [25].

$$P=\frac{1}{\pi }\sqrt[{3}]{\frac{6F}{{\left(\frac{1-\mu_{1}^{2}}{E_{1}}+\frac{1-\mu_{2}^{2}}{E_{2}}\right)}^{2}R^{2}}}$$

### 2.7. Drug Loading and Release

MSNs (20 mg) were added to 10 mL of RhB solution (0.5 mM) in a flask, and dispersed uniformly through ultrasound. Afterwards, the mixture was stirred at room temperature for 24 h, and the RhB-loaded MSNs were separated by centrifugation (8000 r/min, 5 min), washed thoroughly with deionized water, and finally dried under vacuum overnight. Subsequently, these RhB-loaded MSNs were employed to prepare RhB-loaded MSNs@lip according to the procedure as mentioned in Section 2.4. The loading capacity of MSNs, MSNs@lip-1 and MSNs@lip-2 was calculated according to the following equation.

(1) $$loading\;capacity\;\left(\%\right)=\frac{amount\;of\;loaded\;RhB}{amount\;of\;RhB-loaded\;nanoparticles}\times 100$$

The in vitro release profile of RhB from MSNs, MSNs@lip-1 and MSNs@lip-2 was conducted through dialysis tube diffusion at 37 °C for 70 h. Generally, 20 mg of nanoparticles were ultrasonically dispersed in 10 mL of PBS, and 2 mL suspension was placed into a dialysis tube (molecular weight cutoff: 8000–10,000). Subsequently, the tube was dialyzed against 20 mL of PBS at 37 °C. After a predetermined time, 2 mL of the medium was taken out from the release buffer and replaced by 2 mL of fresh PBS solution. The amount of RhB released from MSNs, MSNs@lip-1 and MSNs@lip-2 was evaluated by a UV-Vis spectrophotometer (UV-6100s, Metash Instruments, Shanghai, China), and the drug release profile was drawn accordingly.

### 2.8. In Vitro Cytotoxicity

Cell cytotoxicity of phospholipid-coated MSNs was evaluated by CCK-8 assay using MC3T3-E1 cells. The MC3T3-E1 cells were seeded in a 96-well plate at a density of 3 × 10^4^ cells/well and then incubated in alpha-MEM culture medium supplemented with 1% penicillin-streptomycin for 24 h. Afterwards, the cells were exposed to MSNs, MSNs@lip-1 and MSNs@lip-2 (1 mg/mL) and further incubated for 1 day, 3 days and 5 days. Finally, the CCK-8 working solution was added to the culture medium (100 µL/well), and the cells were incubated for another 3 h. The absorbance was recorded using a microplate reader (Varioskan Flash, Thermo, Waltham, MA, USA) at 450 nm.

## 3. Results and Discussion

### 3.1. Characterization of Phospholipid-Coated MSNs

**Figure 1:**
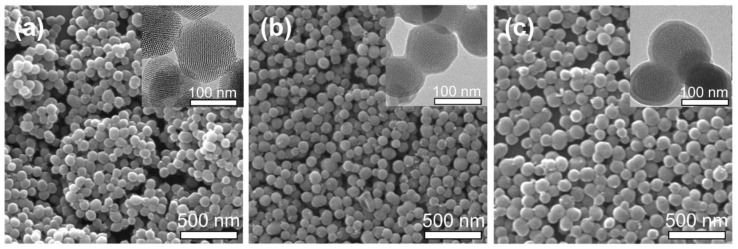
Morphological characterization of MSNs, MSNs@lip-1 and MSNs@lip-2: (**a**) SEM and TEM images of MSNs; (**b**) SEM and TEM images of MSNs@lip-1; (**c**) SEM and TEM images of MSNs@lip-2.

**Figure 2:**
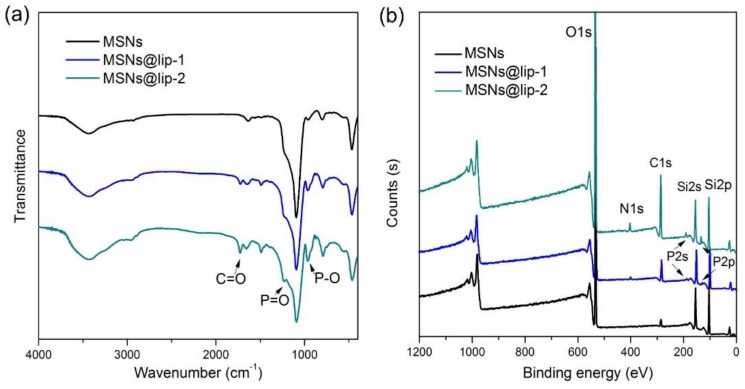
Composition characterization of MSNs, MSNs@lip-1 and MSNs@lip-2: (**a**) FTIR spectra show the presence of P=O and P–O in MSNs@lip-1 and MSNs@lip-2; (**b**) XPS show the presence of P and N elements in MSNs@lip-1 and MSNs@lip-2.

The phospholipid layers were coated onto the surface of MSNs to produce the phospholipid-coated MSNs, i.e., MSNs@lip, which were characterized using SEM, TEM, FTIR, XPS, TGA and DLS. The morphologies of MSNs, MSNs@lip-1 and MSNs@lip-2 examined by SEM and TEM are displayed in Figure 1. MSNs exhibit ordered mesoporous structure, and the nanoparticles are homogeneous and spherical in shape. MSNs@lip-1 and MSNs@lip-2 show clearly the phospholipid layers, which do not result in the blockage of the mesopores in MSNs. The FTIR spectra of MSNs, MSNs@lip-1 and MSNs@lip-2 are presented in Figure 2a. In comparison with MSNs, new absorption peaks are observed in the FTIR spectra of MSNs@lip-1 and MSNs@lip-2. The absorption peaks for P–O and P=O appear at 964 cm^−1^ and 1246 cm^−1^, and the absorption peak at 1730 cm^−1^ corresponds to C=O. The XPS spectra of MSNs, MSNs@lip-1 and MSNs@lip-2 are shown in Figure 2b. For MSNs, the binding energies of Si 2p and Si 2s are 104 and 155 eV, respectively. Compared with MSNs, the new peaks for N 1s, P 2s and P 2p of MSNs@lip-1 and MSNs@lip-2 appear at 398, 189 and 131 eV, respectively. The observation of N, P, P–O and P=O, being solely attributed to DSPC rather than MSNs, indicated that the phospholipid layer was coated onto the surface of MSNs successfully. Additionally, the peak values of N, P, P–O and P=O for MSNs@lip-2 were obviously higher than that of MSNs@lip-1, indicating that the amount of DSPC on the surface of MSNs@lip-2 (multi-layered phospholipid coating) was larger than that of MSNs@lip-1.

**Figure 3:**
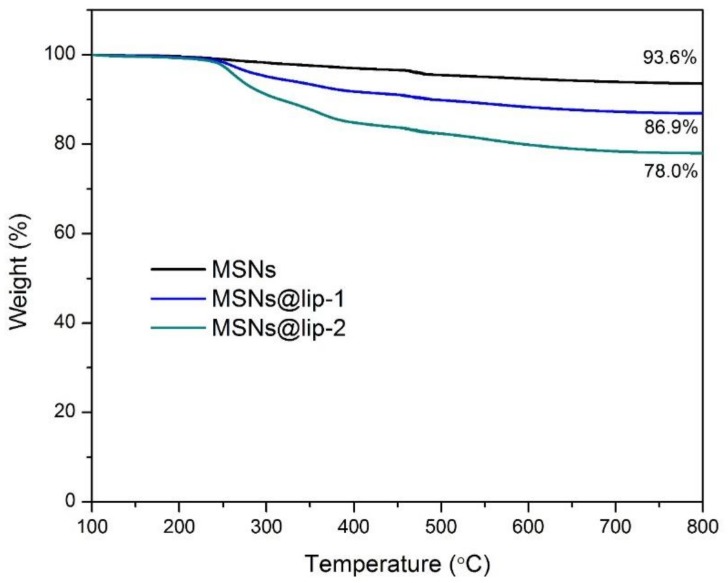
TGA curves of MSNs, MSNs@lip-1 and MSNs@lip-2. The content of phospholipid layer in MSNs@lip-1 and MSNs@lip-2 is calculated to be 7.2% and 16.7%, respectively.

Furthermore, in order to obtain a quantitative evaluation for each component of the phospholipid-coated MSNs, TGA of MSNs, MSNs@lip-1 and MSNs@lip-2 was conducted and the result is demonstrated in Figure 3. The weight loss of MSNs is 6.4%, and after coating of phospholipids layer, the weight loss of MSNs@lip-1 and MSNs@lip-2 increases to 13.1% and 22%, respectively. Accordingly, the content of DSPC in MSNs@lip-1 and MSNs@lip-2 was calculated to be 7.2% and 16.7%, which further confirmed the successful coating of phospholipid layer onto the surface of MSNs.

The average hydrodynamic diameter and Zeta potential of MSNs, DSPC liposomes, MSNs@lip-1 and MSNs@lip-2 were examined by DLS, and the results are shown in Table 1. The average diameter of MSNs is 120 nm, and it increases to 162 nm (MSNs@lip-1) and 384 nm (MSNs@lip-2) following phospholipid coating. The zeta potential changes from −14.8 mV (MSNs) to 8.6 mV (MSNs@lip-1) and then to 13.3 mV (MSNs@lip-2) gradually. The value of the zeta potential of MSNs is negative due to the hydration of the hydroxyl group present on the surface, while that of MSNs@lip-1 and MSNs@lip-2 is positive because the head group of DSPC is quaternary ammonium cations, which face and extend towards the aqueous environment in the phospholipid-coated MSNs. This indicates that the phospholipid layers have been coated on the surface of MSNs successfully.

### 3.2. Lubrication Property

**Figure 4:**
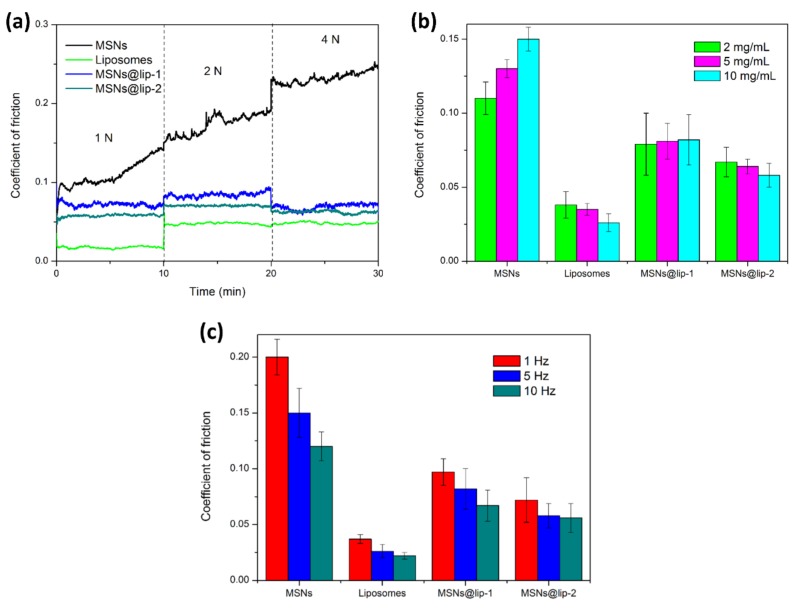
The lubrication property of MSNs, DSPC liposomes, MSNs@lip-1 and MSNs@lip-2 in aqueous suspension evaluated by a series of tribological tests under different conditions: (**a**) friction coefficient-time plots under applied gradient load; (**b**) comparison of the friction coefficient for the nanoparticles at different concentrations; (**c**) comparison of the friction coefficient for the nanoparticles under different reciprocating frequencies.

The result of tribological tests under different conditions for MSNs, DSPC liposomes, MSNs@lip-1 and MSNs@lip-2 is displayed in Figure 4. Figure 4a shows the friction coefficient-time plots for the Ti6Al4V-polyethylene contact pair lubricated by MSNs, DSPC liposomes, MSNs@lip-1 and MSNs@lip-2 under gradient load from 1 N to 4 N. The reciprocating frequency is 3 Hz, and the concentration of the aqueous suspension is 10 mg/mL. It is clear that the friction coefficient of MSNs suspension is significantly higher than that of DSPC liposomes, MSNs@lip-1 and MSNs@lip-2, indicating that MSNs have no lubrication effect when used merely as additive. On the contrary, MSNs may act as abrasive nanoparticles during the tribological tests and result in the enhancement of friction and wear. For DSPC liposomes, the friction coefficient is the smallest under all the applied loads, which is 0.02 under 1 N but increases suddenly to 0.05 under 2 N and remains similar under 4 N. It is considered that the DSPC liposomes in the form of a bilayer can greatly reduce friction due to a shear slip mechanism between the phospholipid bilayers under low load (1 N), but they are crushed under relatively higher load (2 N), resulting in the increase of friction coefficient [26,27]. The friction coefficients of MSNs@lip-1 and MSNs@lip-2 in aqueous suspensions are much smaller than that of MSNs but larger than that of DSPC liposomes, indicating that the phospholipid coated MSNs can act as effective lubricating nanoparticle additives. Under the applied load of 1 N and 2 N, the friction coefficient of MSNs@lip-2 is slightly smaller than that of MSNs@lip-1, which is attributed to the higher content of DSPC, which contributes to enhanced lubrication effect. Likewise, the increase in the friction coefficient under 2 N for MSNs@lip-1 and MSNs@lip-2 is considered to be caused by the crush of the phospholipid coating on the surface of MSNs.

Figure 4b demonstrates the comparison of the friction coefficient lubricated by MSNs, DSPC liposomes, MSNs@lip-1 and MSNs@lip-2 with different concentrations (2, 5, 10 mg/mL). The applied load is 1 N, and the reciprocating frequency is 5 Hz. Obviously, all the friction coefficients of MSNs in aqueous suspension are higher than those of DSPC liposomes, MSNs@lip-1 and MSNs@lip-2, and the value increases gradually with the increase in concentration due to the abrasive effect of MSNs. On the contrary, the value of the friction coefficient decreases gradually with the increase in concentration for DSPC liposomes, which is attributed to the enhanced lubrication effect of phospholipid bilayers. After phospholipid coating, the friction coefficients of MSNs@lip-1 and MSNs@lip-2 decrease considerably in comparison with MSNs but are still larger than those of DSPC liposomes. In addition, it is noted that the value of the friction coefficient for MSNs@lip-2 decreases with the increase in concentration, behaving similarly to DSPC liposomes.

Figure 4c shows the comparison of the friction coefficient lubricated by MSNs, DSPC liposomes, MSNs@lip-1 and MSNs@lip-2 at different reciprocating frequencies (1, 5, 10 Hz). The applied loading is 1 N, and the concentration of aqueous suspension is 10 mg/mL. It is clear that all the friction coefficients decrease with the increase in reciprocating frequency, indicating that the Ti6Al4V-polyethylene contact pair is lubricated under boundary lubrication.

In previous studies, phospholipid-coated MSNs were mainly used in the field of drug delivery [24,28]. To the best of our knowledge, this is the first time that the lubrication property of phospholipid-coated MSNs has been investigated, and the result obtained in the present study indicates that the phospholipid-coated MSNs can promote effective lubrication under typical joint pressure (4–10 MPa) when intra-articularly injected into the joint capsule. Furthermore, the phospholipid-coated MSNs with multi-layers have a lower friction coefficient, which may represent a promising strategy to treat early stage OA by lubrication enhancement and drug delivery therapy. It should be noted that other phospholipids with the phosphocholine head groups can also be used for making the lubricating drug nanocarriers (MSNs@lip), and DSPC is chosen in the present study mainly because the DSPC liposome is featured with higher phase transition temperature and stability.

### 3.3. Drug Loading and Release

**Figure 5:**
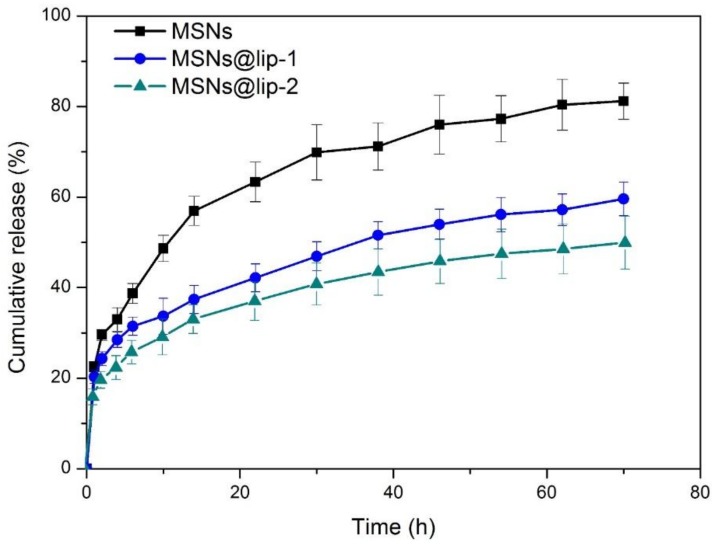
The release profiles of RhB-loaded MSNs, MSNs@lip-1 and MSNs@lip-2 in PBS (pH 7.4) at 37 °C.

The loading capacity of MSNs, MSNs@lip-1 and MSNs@lip-2 is calculated to be 4.9%, 2.8% and 2.6%, respectively. The drug release profiles of RhB-loaded MSNs, MSNs@lip-1 and MSNs@lip-2 are presented in Figure 5. All the curves illustrate an initial rapid drug release within 10 h, followed by a relatively constant phase. After 72 h, 82.3% ± 4.0% of RhB was released from MSNs as the nanocarrier, which is much higher than that of MSNs@lip-1 (59.6% ± 3.7%) and MSNs@lip-2 (50.1% ± 5.9%). Obviously, the amount of RhB released from MSNs is remarkably higher than that of MSNs@lip-1 and MSNs@lip-2, indicating that the phospholipid layer impedes the release of RhB and results in an enhanced sustained release behavior. Additionally, it should be noted that the amount of RhB released from MSNs@lip-2 is less than that from MSNs@lip-1, which is attributed to the multi-layered phospholipid coating on the surface of MSNs. The result of drug loading and release behavior indicates that the phospholipid-coated MSNs can achieve a sustained release of loaded drug when intra-articularly injected into the joint capsule.

### 3.4. In Vitro Cytotoxicity

**Figure 6:**
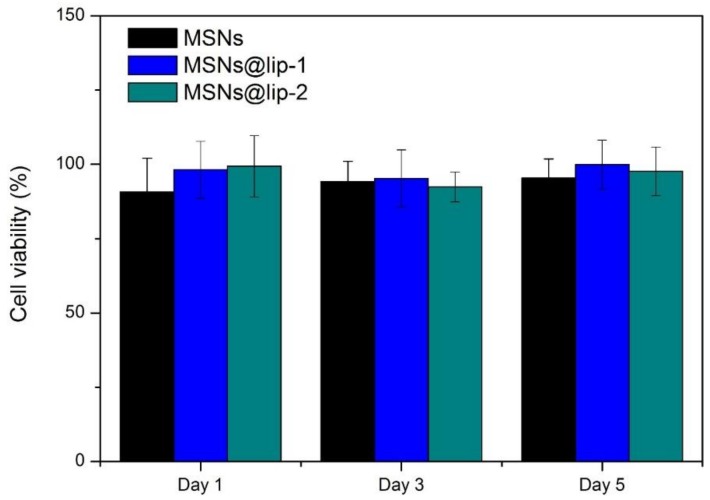
The relative MC3T3-E1 cell viability when incubated with MSNs, MSNs@lip-1 and MSNs@lip-2 for 1 day, 3 days and 5 days.

The in vitro cytotoxicity of MSNs, MSNs@lip-1 and MSNs@lip-2 was evaluated by CCK-8 assay using MC3T3-E1 cells, and the result is shown in Figure 6. Clearly, the relative cell viability of the materials is higher than 80% for all incubation times tested, indicating that the nanoparticles process excellent biocompatibility with the MC3T3-E1 cells. Consequently, it is considered that the phospholipid-coated MSNs are safe for biomedical application.

## 4. Conclusions

In the present study, the phospholipid-coated MSNs were successfully synthesized as effective lubricating drug nanocarriers. The phospholipid layers coated on the MSNs surface achieve excellent lubrication property, while MSNs can load selected drugs and gradually release them afterwards. Additionally, the phospholipid layers on the MSNs surface can inhibit drug release and therefore enable the phospholipid-coated MSNs with sustained release of the drug previously encapsulated in the mesoporous structures of MSNs. Furthermore, the phospholipid-coated MSNs are preferably biocompatible and as a consequence are considered to represent an effective strategy to treat OA where a combination of lubrication enhancement and drug delivery therapy is specifically required.

## Figures and Tables

**Table 1:** The average diameter and Zeta potential of MSNs, DSPC liposomes, MSNs@lip-1 and MSNs@lip-2.

Sample	Average Diameter (nm)	PDI	Zeta Potential (mV)
MSNs	120 ± 12	0.169	−14.8 ± 2.4
DSPC Liposomes	126 ± 27	0.101	12.7 ± 0.6
MSNs@lip-1	162 ± 41	0.204	8.6 ± 1.7
MSNs@lip-2	384 ± 103	0.286	13.3 ± 2.2
